# Additional Note on the Problem of Pyorrhea Alveolaris

**Published:** 1920-12

**Authors:** Stanley Colyer

**Affiliations:** Lond.


### ADDITIONAL NOTE ON THE PROBLEM OF PYORRHEA ALVEOLARIS

BY STANLEY COLYER, M.D.LOND.

The editor of the *Dental Record,* in forwarding me the proof of my paper on Pyorrhea,* asked me how I could explain pyorrhea alveolaris among rice-eating races such as the Indians, Javanese, Chinese and Japanese, and with his permission I am adding this short addendum on the food of the Indians, which may serve to anticipate certain criticisms and strengthen my paper, in the writing of which I intentionally sacrificed detail that the argument might remain clear.

In the first place you can not speak, without misleading others, of a race as rice-eating or maize-eating, as all races are mixed-eating, having certain foods as staple, or customary, and others as additional or seasonal.

### LIST OF FOODSTUFFS OF INDIANS

I. GRAIN.

(*a*) Indian corn.

(*b*) Millets of various kinds, including the ragi (*Eleusine corocana*).

(*c*) Kaffir corn, or great millet.

(*d*) Wheat, barley, buckwheat.

(*e*) Rice.

II. VEGETABLES.

(1) *Above ground.*

(*a*) ————

(*b*) Beans and peas of various kinds.

(*c*) ————

(*d*) An abundance of other vegetables and fruits, such as egg-plant, papaya, banana, etc.

---

* Appeared in our October, 1920, issue.

(2) *Underground.*
   (*a*) Cassava, known in India as manioc.
   (*b*) Monkey-nuts.
   (*c*) Sweet potatoes and allied corms.

III. Flesh Foods.
   (*a*) Oxen, sheep, goats, etc.

IIIa. Oily Foods.
   (*a*) Butter and ghee (melted butter).
   (*b*) Oils expressed from monkey-nuts, coco-nuts, soy-beans, etc.

IV. Savouries and Relish Foods.
   (*a*) Volatile and essential oils and other aromatic compounds contained in garlic, tumeric, pepper, spices, etc.
   (*b*) Sugar in various forms.

V. Drinks.

   (*a*) Alcoholic.

   (*b*) Milk.

   (*c*) Water.

   (*d*) Tea and coffee.

The above list of Indian foodstuffs which I have compiled from Simpson's "Hygiene in Tropical and Sub-Tropical Climates" (1908), is nothing more than a general statement and can not be said to apply definitely to any one section of India. Thus although "rice, ragi, millets, maize and other cereals form as a rule the staple foods of the tropics," Professor Simpson states that "the principal article of food in Bengal, Burmah, Madras, and Assam is rice, mixed with pulse, and a little ghee or melted butter" . . . "whereas in Northern India, which is not a rice-growing country, the millets and pulses are eaten by the poor, while the richer classes are able to afford wheat and barley. Indian corn is eaten by all. There are Hindus who take no animal food beyond that of milk and ghee"—and so on (page 133).

As I am not familiar with Indian life I can not speak with first-hand knowledge either of their foods or habits. Moreover, I do not wish to comment on their diet save to point out its general similarity with that of the African, and to remark that if cassava does not enter largely into their food, the millets, which contain large quantities of gluten, do, and further that sugar and sweetmeats are a national failing.

The thesis of my paper may be said to be that pyorrhea alveolaris is due to soluble food of a viscous nature being held against the gingival margin for a sufficient length of time to lead to damage of the walls of the epithelial cells. Save in a few instances, soluble viscous substances of this nature are not present in the uncooked food of man. They are of course present in uncooked wheat and millets in the form of gluten, and in certain sticky fruits such as dates. Sugar and sweetmeats, especially gelatines and jujubes will, when sucked, give rise to similar sticky and viscous substances. All these are capable of producing the conditions I regard as essential to the production of the disease.

I intentionally contrasted the foods of certain African tribes so that I could clearly show that cassava seemed to be the cause of the disease; but my ambition was to make plain that the cooking of foods leads in many cases to the production of soluble starches which may form a mucilaginous kind of food, and to gelatinous food from the transformation of collagen. That is my generalization; and if it can not explain the geographical distribution of the disease it is incorrect.

I believe dental caries is due to the fermentable carbohydrates, as in sugars and soluable starches. Pyorrhea alveolaris is the result of the additional gelatines formed from callogen, probably by varying processes.—*Dental Record*, Nov., 1920.

